# Genome-wide functional annotation and structural verification of metabolic ORFeome of *Chlamydomonas reinhardtii*

**DOI:** 10.1186/1471-2164-12-S1-S4

**Published:** 2011-06-15

**Authors:** Lila Ghamsari, Santhanam Balaji, Yun Shen, Xinping Yang, Dawit Balcha, Changyu Fan, Tong Hao, Haiyuan Yu, Jason A Papin, Kourosh Salehi-Ashtiani

**Affiliations:** 1 Center for Cancer Systems Biology (CCSB) and Department of Cancer Biology, Dana-Farber Cancer Institute, Boston, MA 02115, USA; 2 Department of Genetics, Harvard Medical School, Boston, MA 02115, USA; 3 Department of Biological Statistics and Computational Biology and Weill Institute for Cell and Molecular Biology, Cornell University, Ithaca, NY 14853, USA; 4 Department of Biomedical Engineering, University of Virginia, Charlottesville, VA 22908, USA; 5 New York University Abu Dhabi, Abu Dhabi, UAE, and Center for Genomics and Systems Biology, Department of Biology, New York University, New York, NY 10003, USA

## Abstract

**Background:**

Recent advances in the field of metabolic engineering have been expedited by the availability of genome sequences and metabolic modelling approaches. The complete sequencing of the *C. reinhardtii* genome has made this unicellular alga a good candidate for metabolic engineering studies; however, the annotation of the relevant genes has not been validated and the much-needed metabolic ORFeome is currently unavailable. We describe our efforts on the functional annotation of the ORF models released by the Joint Genome Institute (JGI), prediction of their subcellular localizations, and experimental verification of their structural annotation at the genome scale.

**Results:**

We assigned enzymatic functions to the translated JGI ORF models of *C. reinhardtii* by reciprocal BLAST searches of the putative proteome against the UniProt and AraCyc enzyme databases. The best match for each translated ORF was identified and the EC numbers were transferred onto the ORF models. Enzymatic functional assignment was extended to the paralogs of the ORFs by clustering ORFs using BLASTCLUST.

In total, we assigned 911 enzymatic functions, including 886 EC numbers, to 1,427 transcripts. We further annotated the enzymatic ORFs by prediction of their subcellular localization. The majority of the ORFs are predicted to be compartmentalized in the cytosol and chloroplast. We verified the structure of the metabolism-related ORF models by reverse transcription-PCR of the functionally annotated ORFs. Following amplification and cloning, we carried out 454FLX and Sanger sequencing of the ORFs. Based on alignment of the 454FLX reads to the ORF predicted sequences, we obtained more than 90% coverage for more than 80% of the ORFs. In total, 1,087 ORF models were verified by 454 and Sanger sequencing methods. We obtained expression evidence for 98% of the metabolic ORFs in the algal cells grown under constant light in the presence of acetate.

**Conclusions:**

We functionally annotated approximately 1,400 JGI predicted metabolic ORFs that can facilitate the reconstruction and refinement of a genome-scale metabolic network. The unveiling of the metabolic potential of this organism, along with structural verification of the relevant ORFs, facilitates the selection of metabolic engineering targets with applications in bioenergy and biopharmaceuticals. The ORF clones are a resource for downstream studies.

## Background

Recent advances in sequencing genomes of prokaryotes and eukaryotes [1] and the explosion of the development and use of genome-scale metabolic network reconstructions [2] are expected to facilitate the selection of targets for metabolic engineering [3,4]] . The unicellular green alga *Chlamydomonas reinhardtii* has been an attractive organism for exploration of metabolic engineering hypotheses due to its capability to flexibly regulate alternative biochemical pathways to produce biofuels [6-9]. However, the optimal selection of the enzymatic targets has been so far hindered by the lack of a comprehensive knowledge of the encoded genes that carry out the metabolic activities of the organism. Although the released genome sequence of *C. renihardtii* by the Joint Genome Institute (JGI) [10] provided the needed resource to predict nearly 17,000 genes in this organism, it alone does not reveal the underlying principles of metabolic network function, nor does it disclose the functions of the predicted “parts-list” of the organism. To define genes and map their products to function, computational algorithms have been extensively applied to annotate the accumulated genomic data from many organisms including *C. reinhartii*[11,12]. Most of these approaches are unable to predict the transcript structures precisely and accurately in a uniform manner due to 1) the incompleteness of the EST data, 2) the lack of comparative genomic information, particularly in less widely studied species, and 3) the complexity of the rules governing transcription initiation, termination and splicing events. Even for the well-studied nematode *C. elegans*, for which a high quality genome sequence has been available for over 10 years, inconsistencies still remain in defining the ORF structures [13,14]]. Previous large-scale studies on *C. reinhardtii*, have included microarray [15,16]], proteomics [17], and, more recently, RNAseq experiments [18] which have provided valuable expression data based on earlier releases of JGI annotations. Currently, the JGI v4.0 predicted *C. reinhardtii* ORFeome remains for the most part unverified; therefore, the functional annotation and experimental structural verification of the encoded ORFs are urgently needed prior to use in functional studies including metabolic engineering experiments.

We previously reported the functional annotation of the gene products involved in central metabolism of *C. reinhardtii* using JGI v3.0 gene models [19] in which we improved the existing functional and structural annotations of the ORF models. In the re-evaluation of the central metabolic ORFs, for which the ORFs are generally the best characterized in the proteome, we observed that as much as 10% of the ORFs were annotated with structuralerrors. The errors included incorrect 5’ or 3’ boundary annotations, which we identified through RACE [19].

In this study, we computationally assigned enzyme functions to the predicted and newly released JGI v4.0 protein-coding ORF models and targeted the enzymatic ORFeome for structural verification. Our results, in addition to structural verification, provide expression evidence for the enzymatic gene products, predict their subcellular localization, and identify the ORF models that may need to be re-annotated.

## Results and discussion

### Functional annotation of JGI v4.0 transcripts

**Figure 1:**
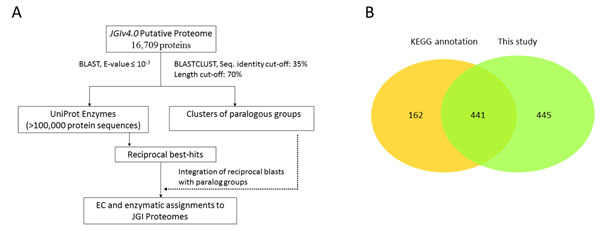
**Functional annotation of *C. reinhardtii* JGI v4.0 translated ORFs.** Enzymatic functions were assigned to the JGI v4.0 translated ORFs by comparing the sequences with the UniProt and AraCyc enzyme databases. The computational pipeline (A) entailed transfer of enzyme annotation to JGI ORFs identified through reciprocal BLAST, then establishing paralog groups to extend enzyme annotation to paralogs. Our functional annotation identified 886 EC numbers, of which only ~50% are currently annotated by KEGG (B).

We used the new JGI “filtered transcript models” released through the JGI portal (http://genome.jgi-psf.org/Chlre4/Chlre4.home.html) for both functional assignments and structural annotation verifications. Enzymatic functional assignments to the *C. reinhardtii* ORFs were made by associating Enzyme Commission (EC) numbers through reciprocal BLAST searches against the UniProt enzyme database [20] (http://www.uniprot.org/, with over 100,000 protein entries) (Figure 1A) supplemented with AraCyc database entries [21] . The best match for each translated ORF was identified (with an e-value threshold of 10^-3^) and the EC number from the UniProt best match (or enzyme annotation from AraCyc) was transferred on to the JGI predicted ORF. We extended the EC assignments to the respective paralogs of the ORFs by clustering ORFs for the JGI filtered models. Altogether, we were able to assign 886 EC numbers to 1,427 JGI ORFs (Figure 1B, Additional file 1). KEGG currently provides 603 enzymatic annotations for the JGI v4.0 transcripts, of which there are 441 shared with our annotation. Theassignments given in this study provide an additional 445 EC numbers not present in KEGG. The list of the enzymatic JGI v4.0 gene models with their assigned EC numbers are provided in Additional file 1.

In order to provide additional functional information, WoLF PSORT [22] was implemented to assign subcellular localizations to each translated JGI v4.0 enzymatic ORF. WoLF PSORT is a high-performance localization prediction algorithm evolved from PSORT [23] , PSORT II [24] and iPSORT [25]; it combines localization features from these algorithms together with amino acid composition in a weighted *k*-nearest neighbors framework. Based on the cross-validation results, WoLF PSORT makes reliable predictions for nucleus, mitochondria, cytosol, plasma membrane, extracellular and (in plants) chloroplast. For other subcellular compartments, the performance is not as good, but still informative [22] . Compared to other methods, WoLF PSORT has been shown to have good performance for most subcellular localizations [26]. Importantly, predictions are not made on the basis of signal sequences that can introduce vulnerability to errors in sequence and/or annotations on the 5' end of the gene [27]. Furthermore, due to the unique phylogenetic position of *C. reinhardtii* and a lack of extensive GO annotation, alternative methods such as MultiLoc2 [28], which use GO annotation for refinement of predictions, would not be applicable here.

**Figure 2:**
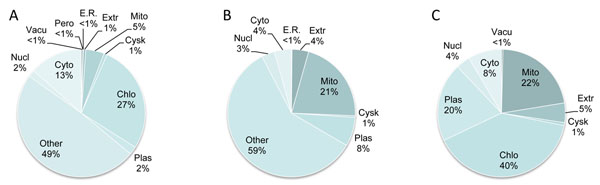
**Subcellular localization prediction of JGI v4.0 enzymes.** Following enzyme classification assignments to JGI v4.0 translated ORFs, subcellular localization of the proteins were predicted by WoLF PSORT [22] as plant (A) or animal (B) proteins. Based on the obtained probability values, each protein was assigned a compartment when 50% or higher percentage of the nearest neighbors for the protein belonged to a given compartment. When the 50% threshold is not reached, the protein, or its encoding ORF are assigned to “other” category to designate multiple compartments or ambiguous predictions. In (C), the predictions made as animal and plant were consolidated into a single set by increasing the threshold to 85%, then reporting the predicted assignment with the higher value. Abbreviations are: Chlo: chloroplast, Cyto: cytosol, Cysk: cytoskeleton, E.R.: endoplasmic reticulum, Extr: extracellular, Mito: mitochondrion, Nuc: nucleus, Pero: peroxisome, Plas: plasma membrane, Vacu: vacuolar membrane.

The results (Additional file 2) are presented as the number of nearest neighbors in different subcellular compartments for each protein. The default value for the total number of nearest neighbors (i.e., *k*) is 32. Even though *C. reinhardtii* is in the plant lineage, it has retained key animal genes [10] and is a unicellular organism that shares ancestry at the branching point of plants and animals. We therefore performed two WoLF PSORT runs in which *C. reinhardtii* was considered either as a plant or animal. Because *C. reinhardtii* is closer to plants than animals [10], predictions made when considering it as a plant are likely to be more accurate. However, because WoLF PSORT uses homology to known proteins, and some *C. reinhardtii* proteins may be closer to those in animals than plants [10], the predictions assuming an animal lineage provide alternative assignments, particularly for cases where ambiguous predictions are made for the proteins assuming plant origins. To summarize the obtained results (Fig. 2), we have binned the encoded proteins based on the assigned probability values for each protein, such that, if more than 50% of the nearest neighbors of the protein belong to a given compartment, that protein is assigned to a single compartment as its primary localization site. In cases where different localization predictions made based on animal and plant assumptions both meet an 85% cutoff, we took the higher confidence prediction as the final localization assignment (Additional file 3). Using this integration scheme, the largest compartment is the chloroplast when *C. reinhardtii* is considered a plant, and the second largest is the mitochondrion (Fig. 2C). These localization predictions agree with the fact that these genes are all related to metabolism. To verify the performance of our predictions, we manually curated a number of experimentally derived *C. reinhardtii* subcellular protein localizations recently reported by Weinkoop et al.[29]. Due to the limited number of localizations that could be transferred to v4.0 annotations from this study, we were only able to evaluate 9 ORFs in our set. Our predicted localizations of all 9 ORFs agreed with the experimentally determined localizations. Although the number is too small for adequate statistical analysis, it still shows the high quality of the predictions.

### Experimental verification of *C. reinhardtii* enzymatic ORFeome

**Figure 3:**
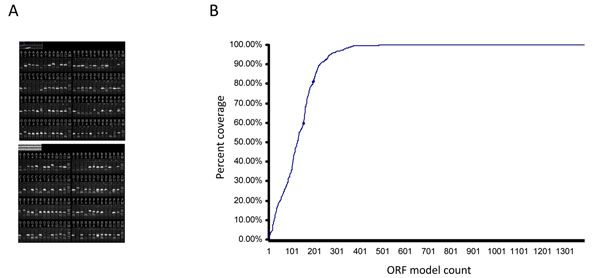
**ORF verification by RT-PCR followed by multiplex sequencing.** RNA isolated from *C. reinhardtii* grown under a permissive condition (continuous illumination and acetate as a source of carbon) was reverse transcribed, then used as template for PCR in which ORF-specific primers were used to amplify the JGI annotated ORFs. The amplicons were then sequenced directly using the 454FLX platform, or cloned, then sequenced by 454. (A) Amplification of representative metabolic ORFs are shown after electrophoresis (192 amplicons analyzed in two 96 well E-gels). (B) Percent coverage of 1,427 enzymatic ORF reference sequences by the obtained reads from 454 sequencing. The 454 reads were aligned to the JGI ORF reference sequences and percent coverage of the length of each reference sequence was determined (100% denotes all bases of the reference sequences could be covered by one or more 454 read). The entire lengths of 699 ORFs were 100% verified.

Our EC annotation of the JGI v4.0 transcript models identified 1,427 predicted transcripts with putative enzymatic functions. To experimentally verify structural annotation of the enzymatic ORFs, we carried out targeted transcriptome sequencing experiments after we amplified the ORFs by reverse transcription-PCR (RT-PCR) (Figure 3A). The generated amplicons were sequenced using the 454FLX platform before and after cloning of the amplicons into a Gateway vector. The sequences of the clones were further verified by conventional Sanger sequencing.

In order to perform the verification experiments, we grew *C. reinhardtii* under permissive condition by providing light, organic carbon sources and other nutrients (Methods). Total RNA from cells undergoing exponential growth was isolated and reverse transcribed to serve as a template for amplification of the ORFs for which we designed Gateway-tailed primers. Following amplification, we carried out next generation sequencing (using the 454FLX platform) of the amplicons. The obtained 454 reads were then aligned to the JGI v4.0 ORF reference sequences to assess annotation accuracy. The aligned ORFs were binned according to their percent coverage; i.e., based on the percentage of the entire length of the ORF reference sequence that could be covered by the contigs assembled from the 454 reads.

For 78% of the JGI v4.0 ORF reference sequences, the 454 reads provided 95-100% coverage (Fig. 3B; Additional file 1), of this set approximately 92% had a coverage rate of 99-100%, demonstrating high verification rates. Approximately 10% of the ORF models showed coverage of 50-95%. The remaining 12% were covered less than 50% and of this set, 7% of the ORF models had less than 20% of their length verified by 454-reads.

As an alternative method of verifying the ORFs, we end-sequenced the cloned PCR products by conventional high-throughput Sanger sequencing. From 1,427 JGI v4.0 ORFs tested, we were able to obtain 661 ORF sequence tags (OSTs) that were aligned to the 5’ end of the ORF models, and 631 OSTs that could be aligned to the 3’ ends. Altogether, 42% (602) ORFs had OSTs that verified both ends of the ORF models. We could assemble full-length contigs for 242 ORFs (Additional file 1).

Overall, we obtained expression evidence for 1,401 of 1,427 ORF models with assigned enzymatic functions based on targeted transcriptome sequencing results and sequencing of the clones, though clearly not all of these ORF models can be considered verified. We consider an ORF model to be verified if 98 to 100% of its reference sequence could be covered by 454-reads, or if a full-length contig generated from Sanger sequencing of an obtained clone completely matched the reference sequence. For 73% of the ORF models, the 454-reads give confirmation at the 98-100% level. Sanger sequencing of the clones could verify an additional 36 ORF models (for which we could assemble contigs using 3’ and 5’ end reads). These models can therefore be considered verified, though it should be noted that even 100% coverage of an ORF model does not exclude the possibility of the presence of exons that were not annotated. The high coverage rates do, however, guarantee that the annotated exons are expressed. Furthermore, incomplete coverage by 454-reads does not necessarily imply inaccurate annotation; in some cases, less than 100% coverage could be the result of low expression level of the transcript and consequently low sequencing depth. We note that due to the amplification of the transcripts, the targeted transcriptome method that we have used is expected to normalize the abundance of the amplicons to a degree.

While end verification by Sanger sequencing can confidently verify the 5’ and 3’ ends, this method provides no information on the internal exon structure of long ORFs (unless internal primer walking [30] is carried out). We also find that the overall success rate of sequencing clones using the Sanger method is significantly lower than the 454 sequencing of amplicons. Cloning bottlenecks, failure to generate contigs due to end reads not covering the internal segments, and random sequencing failures could be among the contributing factors. Direct sequencing of amplicons through 454 or other parallel sequencing methods clearly bypasses these limitations.

## Conclusions

A central challenge in the post-genomic era is the mapping of the genotype-phenotype relationship. For biochemical networks, the functional connections between genotype and phenotype are deciphered through the use of the available high-throughput experimental and computational platforms. Each technology can be used to generate a vast amount of data particular to some aspects of a given biochemical network. Ultimately the gathered data could be used to manipulate the biochemical systems for biotechnological and medical purposes. However, such efforts rest upon the availability of accurate structural and functional annotations, as well as the availability of the biological resources, such as ORF clones. In this study, we have carried out both computational functional annotation and direct experimental verification of structural annotations of JGI v4.0 enzymatic ORFs, which include both metabolic and non-metabolic enzymes. We carried out targeted amplification of the ORFs by RT-PCR and sequenced the products (before and after the cloning) to verify the ORF structures. The approach of using targeted amplification of ORFs offers several advantages over other high-throughput approaches that are not targeted; importantly, it establishes the *cis-*connectivity between the 5’ and 3’ ends of the ORF. Such *cis*-connectivity cannot be established from whole transcriptome sequencing, tiling array analysis or other high-throughput transcriptome survey methodologies (e.g., [18,31-34]). In addition, the generated amplicons can be cloned, as we have done so here, to provide reagents for downstream large- or small-scale experiments, which can be used to define genotype to phenotype maps as well as accomplishing bio-engineering tasks. With an ever-increasing number of organisms whose genome sequences are becoming available (e.g., the diatom *Phaeodactylum tricornutum *[35], the algae *Ostreococcus* Sp. [36] and *Volvox carteri*[37]), the need for structural and functional annotation and their verification is clear. The approach and experiments carried out in this study can be readily extended to other species to facilitate functional annotation and structural verification of their gene models.

## Methods

### Enzyme annotation of JGI v4 Proteome

We assigned Enzyme classification (EC) to the translated JGI v4.0 filtered ORF models (Chlre4_best_transcripts and Chlre4_best_proteins) using UniProt [20] and AraCyc [21] enzyme protein sequences and their EC annotations as the basis. The transfer of enzyme annotations to ORF models involved two main steps: (1) Carrying out and deciphering reciprocal best-hits, if any, for each of the translated JGI ORF models to the UniProt and AraCyc sequences, then transferring the EC from the best-hits UniProt/AraCyc sequences to the corresponding ORF models*.* This transfer was done using BLASTP with an e-value threshold 0.001 [38,39]]; (2) Identification of paralogs, in the entire collection of translated JGI models, of already EC assigned translated ORF models and then transferring their EC annotations to their paralogs as well. This transfer was done using BLASTCLUST (http://www.ncbi.nlm.nih.gov/IEB/ToolBox/C_DOC/lxr/source/doc/blast/blastclust.html) with a sequence identity cut-off of 35% and length cut-off of 70%. BLASTCLUST can cluster protein sequences (using BLAST) systematically through pair wise alignments when statistically significant matches are found. Importantly, BLASTCLUST uses “single-linkage” clustering, which allows linkage of clusters through their “best matching” components. This aspect of the algorithm allows for clustering of sequences, which otherwise may lie below a set similarity threshold among themselves, but are linked through a sequence that has an above threshold similarity.

### Subcellular localization predictions

WoLF PSORT [22] was used to assign subcellular localizations to each translated JGI v4.0 enzymatic ORFs. The output for each ORF provides the number of nearest neighbors in different subcellular compartments for each protein. The default value for total number of nearest neighbors (i.e., *k*) is 32. For each protein, the result can be transformed into a probability model:

where *c_i_* is the *i*th subcellular compartment; *N*(*c_i_*) is the number of nearest neighbors the protein has for the *i*th subcellular compartment, and *m* is the total number of subcellular compartments predicted for the protein. We carried out the localization assignments of *C. reinhardtii* ORFs considering it as a plant and animal.

### *C. reinhardtii* strain and growth condition

*C. reinhardtii* strain CC-503 was used for our experiments. *C. reinhardtii* cells were grown in Tris-acetate-phosphate (TAP) medium containing 100 mg l^-1^ carbamicillin without agitation, at room temperature (22–25 °C) and under continuous illumination with cool white light at a photosynthetic photon flux of 60 μmol m^-2^ s^-1^.

### RNA isolation and quality assessment

Total RNA was isolated from *C. reinhardtii* cells grown in TAP medium and under constant light. Cells from mid-log phase were collected by centrifugation at 2,000 rpm (650g) for 10 min. Total RNA was isolated using TRIzol reagent (Invitrogen). The quality of the isolated RNA was improved by digesting the remainder of the cellular DNA using 0.08 U µl^-1^ RNase-free DNase I enzyme (Ambion). The integrity and quality of the total RNA was assessed by Agilent 2100 Bioanalyzer (Agilent) using RNA pico 6000 kit and by following the manufacturer’s instruction. The fraction of RNA with RNA Integrity Number (RIN) of more than 7.5 was used for cDNA synthesis. The concentration of the RNA was measured spectrophotometrically.

### Structural verification of the JGI v4.0 transcripts: Reverse transcription-PCR of the metabolic ORFs

The annotated metabolic ORFs were subjected to reverse transcription followed by PCR to verify their predicted sequences. Reverse transcription of RNA was carried out using Superscript III reverse transcriptase (Invitrogen) following the manufacturer’s instructions using random N6 and dT(16) (Ambion) as universal primers. The reaction mixture contained 1.2 M betaine (Sigma-Aldrich) to prevent premature terminations owing to the high G+C content of the *C. renhardtii* transcriptome. The synthesized cDNAs were used as templates in PCR reactions. ORF-specific primers tailed with Gateway compatible sequences were designed automatically using the OSP program [40] The forward primer starts from nucleotide A of the ATG start codon and was flanked with the Gateway B1.1 sequence at its 5’ end. The reverse primer starts from the codon immediately before the termination codon and carried the Gateway B2.1 sequence at its 5’ end. All primers had a melting temperature (Tm) between 55 °C and 65 °C. KOD hot start DNA polymerase (Novagen) catalyzed the amplification of ~1,430 ORFs individually in separate 50 µl reaction mixtures containing 1.2 M betaine and 0.25 µg/µl cDNA.

### Gateway cloning of the metabolic ORFs, their transformation and amplicon generation for sequencing

The generated amplicons were recombinationally cloned into the pDONR223 Gateway vector to generate Entry clones [41]. The recombinational cloning was performed using BP clonase (Invitrogen) following the manufacturer’s instructions. The Entry clones were subsequently transformed into chemically competent *E. coli* DH5α. The positive transformants were selected and grown in 96-well format plates containing LB and 100 mg/l spectinomycin. Following growth in liquid media, the transformed bacteria were used as a source of template in PCR reactions containing 1.2 M betaine and KOD hot start DNA polymerase (Novagen) to amplify the clones. Vector primers were used to generate the final DNA templates for sequencing.

### Generation of ORF sequence tags (OSTs) by Sanger sequencing

PCR products were sequenced bi-directionally using conventional automated cycle sequencing to generate ORF sequence tags (OSTs) [42]. Sequencing was carried out by Agencourt Bioscience Corp.

Forward and reverse sequences were vector-clipped (using Cross_match, http://www.phrap.org/phredphrap/general.html), then assembled. We used Phrap (http://www.phrap.org/) to assemble the forward and reverse sequences. Both assembled contigs and singlets were aligned against the coding sequences (CDSs) of corresponding predicted transcripts from *C. reinhardtii* assembly v4.0 (http://genome.jgi-psf.org/Chlre4/Chlre4.home.html) using MUSCLE [43,44]]. The alignment files were then used to verify the CDSs of the predicted transcripts. An ORF model was considered verified if a contig could be assembled from both end reads and if the contig verifies the predicted sequence.

### ORF model verification by 454FLX sequencing

The generated ORF amplicons were sequenced using the 454FLX Titanium sequencing system (454 Life Sciences Corp., Roche). For targeted transcriptome sequencing, the amplicons generated in RT-PCR reactions were pooled in equimolar ratios. For verification of cloned ORFs, the PCR products of the entry clones were pooled in equimolar quantities. The resulting mixes were partially purified using Qiagen MinElute PCR purification kit following the manufacturer’s instruction. Five micrograms of DNA from each sample was subjected to nebulization for 90 seconds under nitrogen gas pressure of 30 psi(2.1 bar). After purification of the sheared DNA using the MinElute PCR purification kit, the DNA fragments were size-selected using AMPure beads (Agencourt). DNA fragments with the size range of 300-800 bp were end repaired and the adaptors were ligated to the ends. After melting into single stranded DNA molecules, the quality of the DNA library was assessed on a BioAnalyzer RNA Pico 6000 LabChip (Agilent). The resulting single stranded DNA libraries were then purified and used to set up emulsion PCR reactions according to the manufacturer’s instruction (454 Life Sciences Corp., Roche). After the amplification step, the emulsions were chemically broken and the beads carrying the amplified DNA library were recovered and enriched. The sequencing was performed on the Roche 454 Genome Sequencer Instrument with the GS FLX Titanium Sequencing Kit XLR70. Approximately 800,000 DNA-carrying beads along with enzyme and packing beads were loaded onto a PicoTitrePlate device. The sequencing was operated and monitored for ~9 hrs during which 200 flow cycles were completed. The generated data were processed using the GS FLX data analysis software v2.3. The vector sequences and Gateway tail sequences were trimmed from the raw reads and the reads shorter than 20 nt were filtered out. The trimmed and filtered reads were aligned against JGI v4.0 reference sequences using the GS Reference Mapper application (*gsMapper* v2.3). A minimum overlap length of 40 nt and minimum overlap identity of 90% were used to align the reads against the JGI v4.0 reference sequences. An ORF model was called verified if more than 98% of its entire length was covered by (matched to) the assembled contigs from the 454 reads.

## List of abbreviations used

ORF: Open Reading Frame; OST: ORF Sequence Tag; JGI: Joint Genome Institute

## Authors’ contributions

LG designed the cloning experiments, carried out molecular cloning, 454 sequencing, sequence analysis and drafted the manuscript. SB designed the functional annotation pipeline and carried out functional annotations of the ORFs; DB contributed to cloning; XY contributed to 454 sequencing. YS, CF, and TH carried out primer design and sequence alignments. HY carried out localization prediction of the ORFs. HY, JP, and KSA conceived the study, participated in its design and helped to draft the manuscript. All authors read and approved the final manuscript.

## Competing interests

The authors declare that they have no competing interests.

## Supplementary Material

Additional File 1 JGIv4.0 gene model names, their predicted sequence, EC annotation, and verification status of their structural annotation. Click here for file

Additional File 2 Subcellular localization prediction of JGI v4.0 enzymes predicted by WoLF PSORT as plant or animal proteins. Click here for file

Additional File 3 A consolidated set of high confidence subcellular localization predictions made by WoLF PSORT. Subcellular compartments predicted for JGI v4.0 as plant or animal at 0.85 or higher ratio relative to other compartments were selected then consolidated by reporting the prediction with the higher value. Click here for file
